# Correlates of Untreated Hypercholesterolemia in Older Adults: A Community-Based Household Survey in China

**DOI:** 10.1371/journal.pone.0131318

**Published:** 2015-07-10

**Authors:** Zhi Hu, M. Justin Zaman, Jingjing Wang, Janet L. Peacock, Ruoling Chen

**Affiliations:** 1 School of Health Administrations, Anhui Medical University, Hefei, China; 2 James Paget University Hospital, Gorleston-on-Sea, Norfolk, United Kingdom; 3 Norwich Medical School, University of East Anglia, Norwich, Norfolk, United Kingdom; 4 Division of Health and Social Care Research, King’s College London, London, United Kingdom; 5 NIHR Biomedical Resaerch Centre at Guy’s and St Thomas’ NHS Foundation Trust and King’s College London, London, United Kingdom; 6 Centre for Health and Social Care Improvement, Faculty of Education, Health and Wellbeing, University of Wolverhampton, Wolverhampton, United Kingdom; 7 Post Graduate Academic Institute of Medicine, University of Wolverhampotn, Wolverhampton, United Kingdom; University of Perugia, ITALY

## Abstract

Hypercholesterolemia is common in older adults and less treated, but little is known about correlates of untreated hypercholesterolemia. Using a standard interview method we examined a random sample of 7,572 participants aged ≥60 years in a community-based household survey across 7 provinces of China during 2007–2012, and documented 328 cases of hypercholesterolemia from self-reported doctor diagnosis. Compared to participants with normal cholesterol, older adults with hypercholesterolemia had higher socioeconomic position and larger body mass index. In patients with hypercholesterolemia, 209 were not treated using lipid-lowering medications (63.7%, 95% confidence interval (CI) 58.5%–68.9%). Untreated hypercholesterolemia was significantly associated with female sex (adjusted odds ratio 2.13, 95%CI 1.17–3.89), current smoking (3.48, 1.44–8.44), heavy alcohol drinking (3.13,1.11–8.84), chronic bronchitis (2.37,1.14–4.90) and high level of meat consumptions (2.85,1.22–6.65). Although having coronary heart disease exposed participants for treatment, half of participants with coronary heart disease did not receive lipid-lowering medications. Among hypercholesterolemia participants with stroke, hypertension or diabetes, more than half of them did not receive lipid-lowering medications. The high proportion of untreated hypercholesterolemia in older, high-risk Chinese adults needs to be mitigated through multi-faceted primary and secondary prevention strategies to increase population opportunities of treating hypercholesterolemia.

## Introduction

Hypercholesterolemia is a major risk factor for cardiovascular disease. Clinical trials have repeatedly demonstrated that lipid-lowering medication, mainly in the form of statins, reduces future cardiovascular events. [1,2] This treatment effect is also true for older adults. [3] Serial health surveys conducted in European populations have documented a significant increase in the use of lipid-lowering medication in the general populations over time.[4,5] However, a substantial proportion of people with hypercholesterolemia remain untreated. [6,7] The reason remains unclear. Previous studies have shown that not receiving lipid-lowering medication for primary prevention is associated with younger age, male sex and smoking, [8] while for secondary prevention it is associated with older age and female sex. [9] These studies focused on young and middle aged populations, and the correlates of untreated hypercholesterolemia in older adults have not been well investigated.[10]

Current knowledge about the management of lipid lowering medication in people with hypercholesterolemia is predominately derived from studies undertaken in high-income countries, [7] [8] [9] thus limiting translation of findings to low and middle- income countries (LMICs) across the world. In LMICs, the number of people with hypercholesterolemia has increased over the last three decades, as countries undergo epidemiological transition. [11,12] Studying a population in LMICs may offer internationally applicable insights into the proportion and correlates of untreated hypercholesterolemia. China is the biggest among the LIMCs, and the world’s most populous nation. Since the economic reform of the 1980s, China has had a rapid growth in income and increase in life expectancy. The number of older people in China, defined age of > = 60 years, [13] has increased in the past 3 decades. There is concomitantly an increasing prevalence of hypercholesterolemia in the Chinese population,[14] ensuing coronary heart disease and stroke.[6] In this paper we examine data from a community-dwelling based study of older adults in China [15] to identify proportion and correlates of untreated hypercholesterolemia.

## Methods

The data were derived from a large-scale cross-sectional study in 2007–2011 across 7 provinces in China for this study. [15, 16]

### Study populations

We included all participants in a four-province study, the Hubei province study and the Anhui province cohort 3^rd^ wave study [15], and also Xinjiang Uyghur Autonomous Region study (rural areas) [16]. These studies were community-based household surveys, with a common research protocol which is derived from that in the four-province study [15]. Recruiting participants and collecting data were carried out in 2007–2011. *The Four-Province Study*: The sampling methods of the four-province study population have been fully described before. [15] In brief, we selected one rural and one urban community from each of four provinces (Guangdong, Heilongjiang, Shanghai and Shanxi–four research centres) as the study fields, aiming to recruit more than 500 participants in each community. We employed a cluster randomised sampling method to choose residential communities from each of the four provinces. The target population consisted of residents aged ≥60 years living in the area for at least five years. Ethical approval for the study was obtained from the Research Ethics Committee, University College London, UK, and from the Research Ethics Committee of Anhui Medical University and the local governments in China. Based on the residency list of the committees of the village and the district, we recruited a total of 4,314 participants with an overall response rate of 93.8%. *The Hubei and Xinjiang studies*: we extended the project to include in Hubei province and the Xinjiang Uyghur Autonomous Region, using the same protocol as in the four-province study. In Hubei, we selected one urban and one rural areas to recruited 1,001 participants aged ≥ 60 years, and achieved a response rate of 91.8%. [15] In Xinjiang Uyghur Autonomous Region, we interviewed 500 participants in rural areas in Urumqi County, Lop county and Hami back to the urban and rural for this study, with a response rate of 91.3%. [16] *The Anhui province cohort study—third wave survey*: the methods of the Anhui cohort study has been fully described in previous publications.[15] In brief, in 2001–2003 we interviewed a random sample of 3,336 residents aged ≥ 60 years in urban and rural of Anhui province for mental health in older Chinese (*wave 1*). One year after the baseline investigation we re-examined 2,608 cohort members (*wave 2*). In 2007–2009 we successfully interviewed 1,757 survivors (*wave 3*), with a response rate of 82.4% of surviving cohort members.

### Interview and data collection

Two researchers from each centre (province) team were trained at the Anhui Medical University, where we had previously completed several surveys of mental illness in older people and had an experienced interview team.[17] The trained researchers cascaded skills to local research teams and trained the interviewers. The local survey teams from Anhui, Guangzhou (Guangdong), Harbin (Heilongjiang), Shanxi and Hubei Medical Universities, School of Public Health of Fudan University (Shanghai), and The People's Hospital of Xinjiang Uyghur autonomous region interviewed the participants at home. Permission for interview and written informed consent were obtained from each participant or if that was not possible, from the closest responsible adult. Refusals were respected. In about 5% of the interviews, informed consent was impossible to elicit; in these cases, the nearest relative or carer was approached to provide assent to participation. The main interview included a general health and risk factors record,[17] the Geriatric Mental State (GMS) questionnaire,[18] and other components of the 10/66 algorithm dementia research package.[19]

In the general health and risk factors component we recorded details relating to socio-demography, social networks and support, and cardiovascular (CVD) and risk factors. Socioeconomic variables included rural/urban domicile area, educational level, occupational class, and annual personal and family incomes. Information about hobbies, physical activity and Daily Life Activity were documented. We asked participants (or their carers if the participant was unable to answer) if they had received a doctor’s diagnosis of heart disease (1.coronary heart disease–CHD, 2. valve disease, or 3. others), angina, stroke, diabetes, chronic bronchitis, chronic kidney disease, cancer, epilepsy, Parkinson's disease, etc. We included a dietary intake questionnaire in the general health and risk factors record. We measured systolic and diastolic blood pressure, height, weight and waist circumference for all participants according to standard procedures. [20] The GMS questionnaire data were analysed by a computer program-assisted diagnosis, the Automated Geriatric Examination for Computer Assisted Taxonomy (AGECAT), to assess the principal mental disorders in the study participants. [18]

In the interview, we asked each participant for details of hypercholestoralemia. Participants with hypercholesterolemia were those who gave a positive answer to “*Do you have a doctor-diagnosed hypercholesterolemia*?*”*. If yes, they were asked how many years they had hypercholesterolemia and whether they took any lowering-lipids medication. We defined ‘untreated hypercholesterolemia’ as those who gave negative answers to the last enquiry.

### Statistical analysis

The SPSS statistical package (Windows version 21.0; SPSS Inc, Chicago, Ill) was used for data analysis. Patient characteristics comparing treated and untreated using lipid-lowering medications were tested by chi-square test. Using a logistic regression model we calculated odds ratios (ORs) and 95% confidence intervals (CIs) for untreated hypercholesterolemia in relation to individual characteristics, adjusted for age, sex and duration of hypercholesterolemia. Previous studies showed that age and sex were associated with taking lowering-lipid medications. [8, 9] In China, some older adults would not like to take medications immediately following the diagnosis of a chronic condition, which is not acute and serious. However in time they will eventually take medications for treatment. The time since hypercholesterolemia diagnosis would be also associated with other variables such as the consumption of meat and CHD. Therefore we took these 3 variables for adjustment in an initial logistic regression analysis. We employed a multivariate logistic model, which included urban-rural living and all variables with a p value < = 0.1 in the above adjustment analysis, to assess the correlates for untreated hypercholesterolemia. We carried out a sensitivity analysis in participants who did not have chronic kidney disease (CKD) as the CKD may prevent from using lipid-lowering medications due to its metabolic issues. In the multivariate analysis we further used a stepwise method (forward: Wald) in the logistic model to examine the correlates.

## Results

Of 7,572 participants, 328 (4.3%) were reported to have doctor-diagnosed hypercholesterolemia. Of these with hypercholesterolemia, the average age was 71.4 years (SD 7.1) with a range of 60–93, and 60.1% were women. They are not significantly different from those who were not reported to have hypercholesterolemia (age 72.0 (7.7), 54.9% women). However, they were more likely to have higher levels of education (> = high secondary school 34.1% versus 12.2%) and family income (RMB Yuan 17242 versus 11150), live in an urban area (79.6% versus 42.6%) and have a larger body mass index (BMI at 24.2 versus 22.8).

Among 328 participants with hypercholesterolemia (S1 Dataset included in Supporting Information), 209 were not treated (63.7% (95% CI 58.5%-68.9%)). The odds ratios for receipt of treatment for hypercholesterolemia stratified by individual characteristics in older Chinese adults are depicted in Table 1. Compared to those with treated hypercholesterolemia, patients who were untreated were more like to be current smokers, heavy alcohol drinkers, consume more meat, and have a shorter duration (<3years) of diagnosed hypercholesterolemia. Untreated hypercholesterolemia was inversely associated with coronary heart disease, but it was not significantly associated with other cardiovascular disease and risk factors in Table 1.

**Table 1:** Characteristics of participants with hypercholesterolemia: frequencies and odds ratios for receipt of treatment for hypercholesterolemia in older adults.

	*Treated*		*Untreated*			*Odds ratio adjusted for age*, *sex and duration of hypercholesterolemia*		
Variable	n = 119	%	n = 209	%	*Chi-square test p*	OR†	95%CI	*P* *
**Basic chraracteristics**								
**Age (years)**								
60~64	28	23.5	44	21.1	*0*.*060*	1.11	(0.60–2.06)	
65~74	42	35.3	101	48.3		1.75	(1.03–2.96) ^@1^	
≥75	49	41.2	64	30.6		1.00		*0*.*089*
**Sex**								
Men	52	43.7	79	37.8		1.00		*0*.*495*
Women	67	56.3	130	62.2	*0*.*294*	1.18	(0.74–1.89)	
**Body mass index (kg/m** ^**2**^ **)**								
< 25	80	68.4	131	63.6	*0*.*685*	1.00		*0*.*717*
25–30	28	23.9	57	27.7		1.24	(0.72–2.13)	
≥30	9	7.7	18	8.7		1.19	(0.50–2.83)	
**Socio-economic status**								
**Educational level**								
> = High Secondary Sch.	40	33.6	72	34.4	*0*.*291*	1.00		*0*.*369*
Secondary school	30	25.2	38	18.2		0.68	(0.36–1.29)	
< = Primary Sch.	49	41.2	99	47.4		1.04	(0.61–1.78)	
**Main occupation**								
Official/teacher	46	38.7	65	31.1	*0*.*117*	1.00		*0*.*268*
Businessmen, housewives and other	15	12.6	44	21.1		1.82	(0.87–3.83)	
manual labour or Peasant	58	48.7	100	47.8		1.11	(0.66–1.87)	
**Annual personal income (RMB, Yuan)**								
≥20000	44	37.0	82	39.2	*0*.*750*	1.00		*0*.*438*
≥10000-<20000	54	45.4	86	41.1		0.70	(0.41–1.21)	
<10000	21	17.6	41	19.6		0.86	(0.44–1.70)	
**Averaged income of family member per year (RMB, Yuan)**								
≥20000	38	31.9	66	31.6	*0*.*982*	1.00		*0*.*867*
≥10000-<20000	57	47.9	99	47.4		0.88	(0.52–1.51)	
<10000	24	20.2	44	21.1		0.85	(0.43–1.67)	
**Urban-rurality**								
Urban	99	83.2	162	77.5	*0*.*220*	1.00		*0*.*334*
Rural	20	16.8	47	22.5		1.35	(0.73–2.49)	
**Lifestyles**								
**Smoking status**								
Never	91	76.5	146	69.9	*0*.*037*	1.00		*0*.*017*
Former	18	15.1	24	11.5		1.21	(0.55–2.62)	
Current	10	8.4	39	18.7		3.27	(1.43–7.44) ^@2^	
**Alcohol drinking/ month**								
No	108	90.8	175	83.7	*0*.*118*	1.00		*0*.*050*
1–9 times	5	4.2	9	4.3		1.43	(0.45–4.62)	
10–90 times	6	5.0	25	12.0		3.32	(1.26–8.77) ^@1^	
**Favourite salt level in dishes**								
Light	52	43.7	98	46.9	*0*.*855*	1.00		*0*.*906*
Middle	42	35.3	70	33.5		0.89	(0.53–1.50)	
Heavy	25	21.0	41	19.6		0.98	(0.53–1.83)	
**Consumption of meat in recent 2 years**								
< Once a week	21	17.6	15	7.2	*0*.*019*	1.00		*0*.*015*
Once a week	39	32.8	64	30.6		2.67	(1.20–5.94) ^@1^	
> = Twice a week and < = once a day	30	25.2	71	34.0		3.61	(1.61–8.08) ^@2^	
> = Twice a day	29	24.4	59	28.2		3.15	(1.38–7.16) ^@2^	
**Consumption of fish in recent 2 years**								
< Once a week	20	16.8	31	14.8	*0*.*142*	1.00		*0*.*146*
Once a week	46	38.7	68	32.5		0.91	(0.45–1.82)	
> = Twice a week and < = once a day	28	23.5	75	35.9		1.69	(0.82–3.49)	
> = Twice a day	25	21.0	35	16.7		0.88	(0.40–1.92)	
**Consumption of egg in recent 2 years**								
< Once a week	11	9.2	25	12.0	*0*.*272*	1.00		*0*.*297*
Once a week	29	24.4	39	18.7		0.63	(0.26–1.50)	
> = Twice a week and < = once a day	28	23.5	66	31.6		1.15	(0.49–2.72)	
> = Twice a day	51	42.9	79	37.8		0.76	(0.34–1.73)	
**Consumption of vegetables in recent 2 years**								
< Once a day	5	4.2	12	5.7	*0*.*749*	1.00		*0*.*569*
Once a day	54	45.4	88	42.1		0.57	(0.18–1.74)	
> = Twice a day	60	50.4	109	52.2		0.65	(0.21–1.99)	
**Consumption of fruits in recent 2 years**								
< Once a day	57	47.9	90	43.1	*0*.*693*	1.00		*0*.*726*
Once a day	51	42.9	97	46.4		1.16	(0.71–1.88)	
> = Twice a day	11	9.2	22	10.5		1.34	(0.59–3.04)	
**Do regular exercise**								
No	75	63.0	125	59.8	*0*.*566*	1.00		*0*.*597*
Yes	44	37.0	84	40.2		1.14	(0.71–1.83)	
**Walk often**								
No	22	18.5	42	20.1	*0*.*724*	1.00		*0*.*661*
Yes	97	81.5	167	79.9		0.88	(0.49–1.57)	
**Social network and support**								
**Religious belief**								
No	94	79.0	171	81.8	*0*.*532*	1.00		*0*.*226*
Yes	25	21.0	38	18.2		0.69	(0.38–1.26)	
**Marital status**								
Married	87	73.1	164	78.5	*0*.*528*	1.00		
Widow	29	24.4	40	19.1		0.72	(0.40–1.28)	*0*.*525*
Divorced/never married	3	2.5	5	2.4		1.00	(0.22–4.49)	
**Living condition**								
Live alone	17	14.3	23	11.0	*0*.*383*	1.00		*0*.*423*
Live with somebody	102	85.7	186	89.0		1.33	(0.66–2.65)	
**Number of children**								
0–1	20	16.8	25	12.0	*0*.*367*	1.00		*0*.*332*
2–3	64	53.8	111	53.1		1.21	(0.61–2.42)	
≥4	35	29.4	73	34.9		1.67	(0.80–3.51)	
**Frequency of visiting children/relatives**								
Daily	26	21.8	37	17.7	*0*.*618*	1.00		*0*.*715*
<Daily and ≥ Monthly	46	38.7	89	42.6		1.29	(0.68–2.44)	
< monthly	47	39.5	83	39.7		1.25	(0.66–2.36)	
**Help available when needed**								
No	9	7.6	22	10.5	*0*.*378*	1.00		*0*.*398*
Yes	110	92.4	187	89.5		0.70	(0.30–1.61)	
**Health status and disease histories**								
**Perception of self-health**								
Good	54	45.4	91	43.5	*0*.*061*	1.70	(0.78–3.70)	
Average	47	39.5	102	48.8		2.35	(1.08–5.11) ^@1^	
Poor	18	15.1	16	7.7		1.00		*0*.*080*
**Duration of hypercholestrolimia (years)**								
<3 years	26	21.8	64	30.6	*0*.*087*	1.89	(1.01–3.52) ^@1^	
3-<10 years	52	43.7	94	45.0		1.41	(0.81–2.45)	
> = 10 years	41	34.5	51	24.4		1.00		*0*.*132*
**Hypertension (BP> = 140/90 mm Hg or taking anti-hytpertensive medication)**								
No	25	21.0	53	25.4	*0*.*374*	1.00		*0*.*410*
Yes	94	79.0	156	74.6		0.79	(0.45–1.38)	
**Coronary heart disease**								
No	79	66.4	167	79.9	*0*.*007*	1.00		*0*.*010*
Yes	40	33.6	42	20.1		0.51	(0.30–0.85) ^@2^	
**Stroke**								
No	107	89.9	186	89.0	*0*.*795*	1.00		*0*.*534*
Yes	12	10.1	23	11.0		1.28	(0.59–2.76)	
**Diabetes**								
No	86	72.3	169	80.9	*0*.*072*	1.00		*0*.*102*
Yes	33	27.7	40	19.1		0.64	(0.37–1.09)	
**Chronic kidney disease (CKD)**								
No	107	184	89.9	88.0	*0*.*605*	1.00		*0*.*659*
Yes	12	25	10.1	12.0		1.19	(0.56–2.52)	
**Chronic bronchitis**								
No	106	89.1	169	80.9	*0*.*052*	1.00		*0*.*094*
Yes	13	10.9	40	19.1		1.81	(0.90–3.61) ^@^	
**Vision problems**								
No	73	61.3	110	52.6	*0*.*127*	1.00		*0*.*115*
Yes	46	38.7	99	47.4		1.46	(0.91–2.33)	
**Hearing problems**								
No	95	79.8	158	75.6	*0*.*380*	1.00		*0*.*390*
Yes	24	20.2	51	24.4		1.28	(0.73–2.25)	
**Depression** ‡								
No	102	85.7	184	88.0	*0*.*751*	1.00		*0*.*726*
Subcase	5	4.2	9	4.3		0.99	(0.31–3.12)	
Case	12	10.1	16	7.7		0.72	(0.32–1.61)	
**Dementia** ‡								
No	113	95.0	193	92.3	*0*.*363*	1.00		*0*.*406*
Subcase/Case	6	5.0	16	7.7		1.52	(0.56–4.13)	

† adjusted for age, sex and duration of hypercholesterolemia **(** *model 1* **)**.

‡diagnosed by the GMS-AGECAT.

*overall p value for each whole factor.

^@^ p< = 0.100 and >0.05

^@1^ p< = 0.05 and >0.01

^@2^ p< = 0.01 and >0.001

^@3^ p< = 0.001

Table 2 shows ORs for the correlates of untreated hypercholesterolemia in a multivariate analysis. Being untreated was significantly associated with being female, current smoking, heavy alcohol drinking and more consumption of meat, and inversely with CHD. The association with chronic bronchitis was borderline significant. In the sensitivity analysis after excluding participants with chronic kidney disease, the findings of the associations were similar to those in the data analysis of all participants, but less significant probably due to a smaller number of participants.

**Table 2:** Multivariate logistic regression^†^ to determine factors associated with untreated hypercholesterolemia in older adults.

	All participants			Participants without CKD		
Variable	OR	95%CI	*P* *	OR	95%CI	*P* *
**Age (years)**						
60~64	1.11	(0.55–2.24)		1.07	(0.51–2.22)	
65~74	1.67	(0.94–2.97)		1.63	(0.89–2.98)	
≥75	1.00		*0*.*183*	1.00		*0*.*242*
**Sex**						
Men	1.00		*0*.*039*	1.00		
Women	1.92	(1.03–3.58) ^@1^		2.01	(1.03–3.90) ^@1^	*0*.*041*
**Urban-rurality**						
urban	1.00		*0*.*530*	1.00		
Rural	1.25	(0.62–2.53)		1.24	(0.61–2.52)	*0*.*557*
**Smoking status**						
Never	1.00		*0*.*055*	1.00		*0*.*127*
Former	1.22	(0.52–2.83)		1.06	(0.44–2.54)	
Current	3.05	(1.22–7.59) ^@1^		2.54	(1.00–6.42) ^@1^	
**Alcohol drinking per month**						
No	1.00		*0*.*040*	1.00		*0*.*051*
1–9 times	0.62	(0.16–2.34)		0.69	(0.18–2.73)	
10–90 times	3.58	(1.22–10.47) ^@1^		3.61	(1.20–10.80) ^@1^	
**Self-feeling health status**						
Good/very good	1.32	(0.57–3.04)		1.55	(0.62–3.85)	
Average	1.88	(0.81–4.36)		2.05	(0.82–5.16) ^@^	
Poor	1.00		*0*.*235*	1.00		*0*.*273*
**Duration of hypercholestrolimia (years)**						
<3 years	1.80	(0.90–3.58) ^@^		1.82	(0.88–3.76) ^@^	
3-<10 years	1.28	(0.69–2.36)		1.51	(0.78–2.91)	
> = 10 years	1.00		*0*.*247*	1.00		*0*.*250*
**Diabetic**						
No	1.00		*0*.*327*	1.00		*0*.*279*
Yes	0.74	(0.41–1.34)		0.70	(0.37–1.33)	
**Coronary heart disease**						
No	1.00		*0*.*007*	1.00		*0*.*023*
Yes	0.46	(0.26–0.81) ^@2^		0.48	(0.26–0.91) ^@1^	
**Chronic bronchitis**						
No	1.00		*0*.*083*	1.00		*0*.*058*
Yes	1.95	(0.92–4.15) ^@^		2.21	(0.98–5.00) ^@^	
**Meat**						
< Once a week	1.00		*0*.*027*	1.00		*0*.*111*
Once a week	2.29	(0.96–5.43) ^@^		1.98	(0.81–4.85)	
> = Twice a week and < = once a day	3.65	(1.53–8.73) ^@2^		2.96	(1.21–7.26) ^@1^	
> = Twice a day	2.97	(1.22–7.22) ^@1^		2.45	(0.96–6.23) ^@^	

† including the variables in model 1, factors with p value <0.10 in Table 1, and variables of urban-rurality and diabetes.

*overall p value for each whole factor.

^@^ p< = 0.100 and >0.05

^@1^ p< = 0.05 and >0.01

^@2^ p< = 0.01 and >0.001

^@3^ p< = 0.001

In the stepwise regression analysis for all patients, the final-step results showed that being untreated was associated with female sex, current smoking, heavy alcohol drinking, CHD, chronic bronchitis and more consumption of meat (Table 3).

**Table 3:** Stepwise logistic regression^†^ to determine factors associated with untreated hypercholesterolemia in older adults.

Variable	OR	95%CI	*P* *
**Sex**			
Men	1.00		*0*.*014*
Women	2.13	(1.17–3.89) ^@1^	
**Smoking status**			
Never	1.00		*0*.*019*
Former	1.17	(0.51–2.67)	
Current	3.48	(1.44–8.44) ^@2^	
**Alcohol drinking per month**			
No	1.00		*0*.*054*
1–9 times	0.58	(0.16–2.10)	
10–90 times	3.13	(1.11–8.84) ^@1^	
**Coronary heart disease**			
No	1.00		*0*.*002*
Yes	0.41	(0.24–0.72) ^@2^	
**Chronic bronchitis**			
No	1.00		*0*.*020*
Yes	2.37	(1.14–4.90) ^@1^	
**Meat**			
< Once a week	1.00		*0*.*021*
Once a week	2.21	(0.98–4.99)	
> = Twice a week and < = once a day	3.61	(1.57–8.31) ^@2^	
> = Twice a day	2.85	(1.22–6.65) ^@1^	

† all variables selected in the analysis were the same as those in Table 2.

*overall p value for each whole factor.

^@^ p< = 0.100 and >0.05

^@1^ p< = 0.05 and >0.01

^@2^ p< = 0.01 and >0.001

^@3^ p< = 0.001

## Discussion

In this community-based health survey of older adults in China we found that about two-thirds of patients who had self-reported doctor-diagnosed hypercholesterolemia were not on lipid-lowering medication. Untreated hypercholesterolemia was associated with female sex, current-smoking, heavy alcohol drinking, chronic bronchitis, and more consumption of meat.

### Strengths and weakness of study

Our study is the first to report correlates of untreated hypercholesterolemia in older adults in China. The studied participants are derived from a multi-province community-based survey and thus the data reflects ‘real-world’ older population. Our study investigated dietary intake factors and physical activities which were not investigated in previous studies. [7] [8] [9] When we examined correlates of untreated hypercholesterolemia, we carried out a sensitivity analysis by excluding participants with chronic kidney disease, a contra-indication to most lowering-lipid medications. Our study is cross-sectional, and thus the causal-result relationships between correlate factors and untreatment require a large-scale cohort study to confirm. We do not have data on serum cholesterol from each patient as the hypercholesterolemia is self reported doctor-diagnosed and this may lead to an underestimate of the prevalence of hypercholesterolemia. A previous national survey in China used a cut-off point of > = 5.72 mmol/L in serum total cholesterol level to produce a 6.1% prevalence of hypercholesterolemia in older adults aged 60 years or over. [21] Its prevalence rate would be closer to our study with its 4.3%, if a cut-off point of > = 6.22 mmol/L were used to define hypercholesterolemia according to the Chinese guidelines for management. [22] We acknowledge that participants with undetected hypercholesterolemia were mostly from lower socioeconomic groups, perhaps as they were less likely to undergo routine health screening, thus potentially resulting in the lack of association we note between socioeconomic deprivation and being untreated. However, missing such participants would not attenuate the significant associations we observe between untreated hypercholesterolemia and other individual characteristics in the analysis. Without data of serum cholesterol level from each patient we were unable to investigate whether the patients with very high hypercholesterolemia took medicines while and those with moderate to low levels of hypercholesterolemia did not. Further research on determinants of untreated hypercholesterolemia in older adults is required.

Data from the World Health Orgnisation (WHO) showed that in China about 230 million people have cardiovascular disease and one in five adult Chinese have a cardiovascular disease. [23] Cardiovascular disease has been listed as one of top priority in China National Plan for NCD Prevention and Treatment (2012–2015).[23] High cholesterol is one of the main risk factors for cardiovascular disease leading to coronary heart disease, stroke and death. In 2007, before our survey started, the Chinese guidelines for prevention and treatment for hypercholesterolemia in adults was published[24] and stated that since the clinical benefits of lowering lipids on CHD incidence are seen for all ages of participants, treatment of hypercholesterolemia in older adults is indicated, and moreover there are no contraindications of the medication treatment. With the higher co-morbidity in older people such as a decline in renal function, however such treatment needs to be personalised and the initial dosage should start low. [24] Apart from lipid-lowering medications, dietary intake control and physical activity also lower lipid levels. However, in the current study there is no evidence that not being on lipid-lowering medications was accounted for by dietary intake control and physical activity. In contrast, not being on lipid-lowering medication among hypercholesterolemia participants was significantly associated with smoking, which is consistent with the findings in previous studies in high income countries.[8] A more interesting finding was that those with untreated hypercholesterolemia had higher levels of alcohol drinking and meat consumption than treated participants. Thus there was no evidence that being untreated was due to other lifestyle interventions for controlling hypercholesterolemia, known to be effective in wider populations. [14]

We did not find that women with hypercholesterolemia had less treatment in the initial analysis. However, in the multivariate analysis including the smoking variable, being untreated was significantly associated with female sex as Chinese women rarely smoke. Sex inequality in treatment has also been found in other studies; in secondary prevention, women had less treatment than men. [9] Our data showed that women with hypercholesterolemia received less treatment than men. This would suggest an important inequality in health care as we found previously in mental health. [15] That hypercholesterolemia participants with chronic bronchitis had double the odds of not being treated with lipid-lowering medication may reflect a polypharmacy issue here, as participants may find it would be hard to cope with multiple medicines.

In this study we observed that more than half of those participants with CHD (51.2%), stroke (65.7%), hypertension (62.4%) or diabetes (54.8%) were not treated using lowering lipid medications (Table 1). Although the finding of a significant association between CHD and treatment for hypercholesterolemia is consistent with those in previous studies, [7] it should be noted that only about half of CHD patients had treated hypercholesterolemia in China. This is much lower than that in high income countries, e.g., 79% of CHD patients were on lipid-lowing medications in the EUROASPIRE III study.[25] Our data further showed that the proportions of participants with hypertension, diabetes and stroke who took lipid-lowing medications were significantly lower than the participants in high income countries.[5,26] These findings highlight that there is lack of awareness of major aetiologic and prognostic factors of cardiovascular disease, and the importance of secondary drug prevention for public health in China.

The low proportion of participants who were on lipid-lowing therapy could be also explained by their belief about the condition and medicines. Most Chinese people feel hypercholesterolemia is not a serious condition because there are no physical symptoms associated with this condition and thus complications may not occur. Furthermore, there is a common perception in the general population in China, particularly amongst older people, that any medicine has a side effect or toxicity, perhaps in part due to stories about the side-effects in Chinese medicine. [27,28] For instance some Chinese consider that in older people, treatment using mediation is not merited because of a perceived lack of prognostic benefit and increased side effects. Therefore participants with hypercholesterolemia are more likely not to take medicine or take it intermittently until the disease progresses and complications appear. This may explain the finding that those with a good perception of self-health may have an increased risk of being untreated for hypercholesterolemia. Further study into health behaviour and belief about medicines would help to ascertain whether behaviour and belief play important roles in patient acceptance of chronic disease medication management. A health promotion program to manage these risk factors for cardiovascular disease and secondary prevention is perhaps part of the plan needed to tackle this growing public health problem.

In *conclusion*, more than two-thirds of older adults with hypercholesterolemia were not on lipid-lowering medication in China. Being untreated was positively related to being female, current smoking, heavy alcohol drinking, more consumption of meat, and chronic bronchitis. These participants with co-morbidities of coronary heart disease, stroke, hypertension or diabetes were given less treatment for hypercholesterolemia compared to those in high income countries. Qualitative research is needed to better understand why this occurs–from doctors, patients and carers. Our research findings highlight a pressing need to tackle untreated hypercholesterolemia in older adults with hypercholesterolemia.

## Supporting Information

S1 Dataset “WJJ 328 RC sent to PLoS copied AAAA.sav” for analysis in this paper. (SAV)
